# *In vitro* and *in vivo* evaluation of self-assembled chitosan nanoparticles selectively overcoming hepatocellular carcinoma via asialoglycoprotein receptor

**DOI:** 10.1080/10717544.2021.1983077

**Published:** 2021-10-01

**Authors:** Rensong Sun, Linlin Fang, Xia Lv, Jiani Fang, Yuting Wang, Dapeng Chen, Liang Wang, Jun Chen, Yan Qi, Zeyao Tang, Jianbin Zhang, Yan Tian

**Affiliations:** a Collage of Pharmacy, Dalian Medical University, Dalian, China; b Collage of Integrative Medicine, Dalian Medical University, Dalian, China; c Laboratory Animal Center, Dalian Medical University, Dalian, China

**Keywords:** Chitosan nanoparticle, hepatocellular carcinoma, asialoglycoprotein receptor, self-assembled, liver targeting

## Abstract

Hepatocellular carcinoma (HCC) is one of the major causes of cancer-related mortality worldwide. Nowadays, liver-targeting drug delivery system has been proven as a promising strategy for overcoming HCC. Asialoglycoprotein receptor (ASGPR) is an ideal receptor for liver targeting, which is mainly expressed on hepatocytes. In this study, we developed several novel liver-targeting chitosan nanoparticles to selectively overcome HCC via ASGPR. Chitosan nanoparticles (Gly-CS-VE, Gal-Gly-CS-VE, Gly-CS-DCA, and Gal-Gly-CS-DCA) were prepared by grafting hydrophilic group (glycidol, Gly), hydrophobic group (deoxycholic acid, DCA or vitamin E succinate, VE), and ASGPR recognizing group (galactose, Gal). Subsequently, their characterizations were measured by ^1^H NMR, FT-IR, TEM, and DLS. Doxorubicin (DOX) was loaded in nanoparticles and released out in a pH-dependent manner. Most importantly, the galactosylated Gal-Gly-CS-VE and Gal-Gly-CS-DCA nanoparticles exhibited significantly stronger *in vitro* cell internalization, cytotoxicity, anti-migration capabilities and *in vivo* anticancer efficacies than the corresponding Gly-CS-VE and Gly-CS-DCA nanoparticles, as well as free DOX. Finally, the four chitosan nanoparticles exhibited good biocompatibility without causing any obvious histological damage to the major organs. Overall, the galactosylated chitosan nanoparticles were proven to be promising pharmaceutical formulations for selectively overcoming HCC, with great potential for clinical applications.

## 1. Introduction

Liver cancer is the sixth most commonly diagnosed cancer and the fourth leading cause of cancer death worldwide in 2020, with approximately 906,000 new cases and 830,000 deaths annually (Sung et al., 2021). Hepatocellular carcinoma (HCC) accounts for 75–85% of primary liver cancer cases. China and eastern Africa are the most high-risk HCC areas, and almost half of HCC patients are in China (Raoul & Edeline, 2020). The causes of HCC are commonly known as hepatitis B virus (HBV) infection, hepatitis C virus (HCV) infection, aflatoxin exposure, heavy alcohol intake, obesity, and type 2 diabetes (Khemlina et al., 2017). Several therapeutic strategies have been applied for HCC treatment, including: (1) hepatectomy, liver transplantation, and percutaneous local ablative therapy for early HCC; (2) transarterial chemoembolization and radiotherapy for intermediate HCC; (3) systemic chemotherapy, targeted therapy, and immunotherapy for advanced HCC (Narsinh et al., 2018). Unfortunately, there are no obvious symptoms at the early stage of HCC, leading them developed to the advanced stage when diagnosed (Dai et al., 2020). As unsuitable for surgical therapy, advanced HCC are commonly treated by chemotherapy. Molecular targeted therapeutic drugs sorafenib and lenvatinib are the current first-line clinical treatment drugs. Traditional anticancer drugs [such as doxorubicin (DOX), 5-fluorouracil, gemcitabine, capecitabine, cisplatin] as well as traditional Chinese medicine (TCM) can also be used for advanced HCC treatment (Ikeda et al., 2018; Liao et al., 2020). However, the therapeutic efficacies of these drugs are unsatisfied, due to some severe problems such as serious side effects, lack of selectivity, multi-drug resistance (Zhang et al., 2019; Xiang et al., 2020). Hence, it is essential to develop a novel strategy to enrich chemotherapeutic drugs in HCC tissue, and prevent their distribution in normal tissue.

In recent decades, nanotechnology has become an attractive strategy for reducing toxicity and enhancing the efficacy of chemotherapeutic drugs. Various nanoparticles as carriers of chemotherapeutic drugs have exhibited an enhanced effect on HCC treatment, including polymer nanoparticles, inorganic nanoparticles, liposomes, microemulsions, nanocapsules, etc (Dong et al., 2017; Zhai et al., 2018; Li et al., 2020; Qi & Liu, 2021). Among various nanoparticles, chitosan nanoparticles attracted many attentions of scientists due to the advantages of good biodegradability and biocompatibility, low toxicity, abundant source, and convenient modification (Bakshi et al., 2020; Bonferoni et al., 2020). Chitosan is a naturally occurring polysaccharide, commonly applied for loading small molecule, protein, or gene drugs. Self-assembly is a common method to prepare chitosan nanoparticles. As chitosan contains amino and hydroxyl groups, amphiphilic chitosan can easily be obtained by grafting hydrophilic groups and hydrophobic groups, which can form self-assembled nanoparticles in aqueous solution. Carboxymethyl, hydroxyethyl, polyethylene glycol, glycidol, and quaternary ammonium groups are generally applied as hydrophilic shell of nanoparticles, while stearic acid, cholesterol, deoxycholic acid, oleic acid, and even lipophilic drugs can be used for hydrophobic core of nanoparticles (Quinones et al., 2018). The self-assembled chitosan nanoparticles can significantly improve the solubility of poorly water-soluble anticancer drugs, such as doxorubicin, paclitaxel, cisplatin, and irinotecan (Zhang et al., 2019). Moreover, chitosan nanoparticles can passively target tumor tissue by the enhanced permeability and retention (EPR) effect. In particular, the positive charge of chitosan could promote the endocytosis of nanoparticles by tumor cells, and escape from endosome by the proton sponge effect (Richard et al., 2013; Vermeulen et al., 2018).

Apart from passive targeting method, chitosan nanoparticle can also actively target tumor tissue by grafting certain ligands. Folic acid, biotin, galactose, hyaluronic acid, transferrin and RGD peptides are frequently reported targeting ligands, which can specifically recognize the overexpressed receptors on tumor cells. Asialoglycoprotein receptor (ASGPR), a transmembrane protein, is considered as an ideal receptor for liver targeting. ASGPR is called galactose receptor or hepatic lectin, because it is mainly expressed on hepatocytes and minimally on extra-hepatic cells (D’Souza & Devarajan, 2015). It can specifically recognize nanoparticles with galactose or *N-*acetylgalactosamine residue, and internalized them via clathrin-mediated endocytosis (D'Souza & Devarajan, 2015; Huang et al., 2018). Galactosylated chitosan (Gal-CS) is a chitosan derivative containing galactose residue, which can be obtained by grafting lactobionic acid onto chitosan. Nanoparticles prepared by Gal-CS have shown great potential in active targeting to liver. Anticancer drugs including gemcitabine, triptolide, doxorubicin, 5-fluorouracil, and siRNA have been loaded in galactosylated chitosan nanoparticles for HCC treatment (Wang et al., 2018; Nair et al., 2019; Zhang et al., 2019; Xiang et al., 2020; Zheng et al., 2020). The results demonstrated that the nanoparticles could significantly enhance the cell uptake, cell apoptosis, and anticancer capabilities of drugs via liver-targeting effect.

**Scheme 1.**
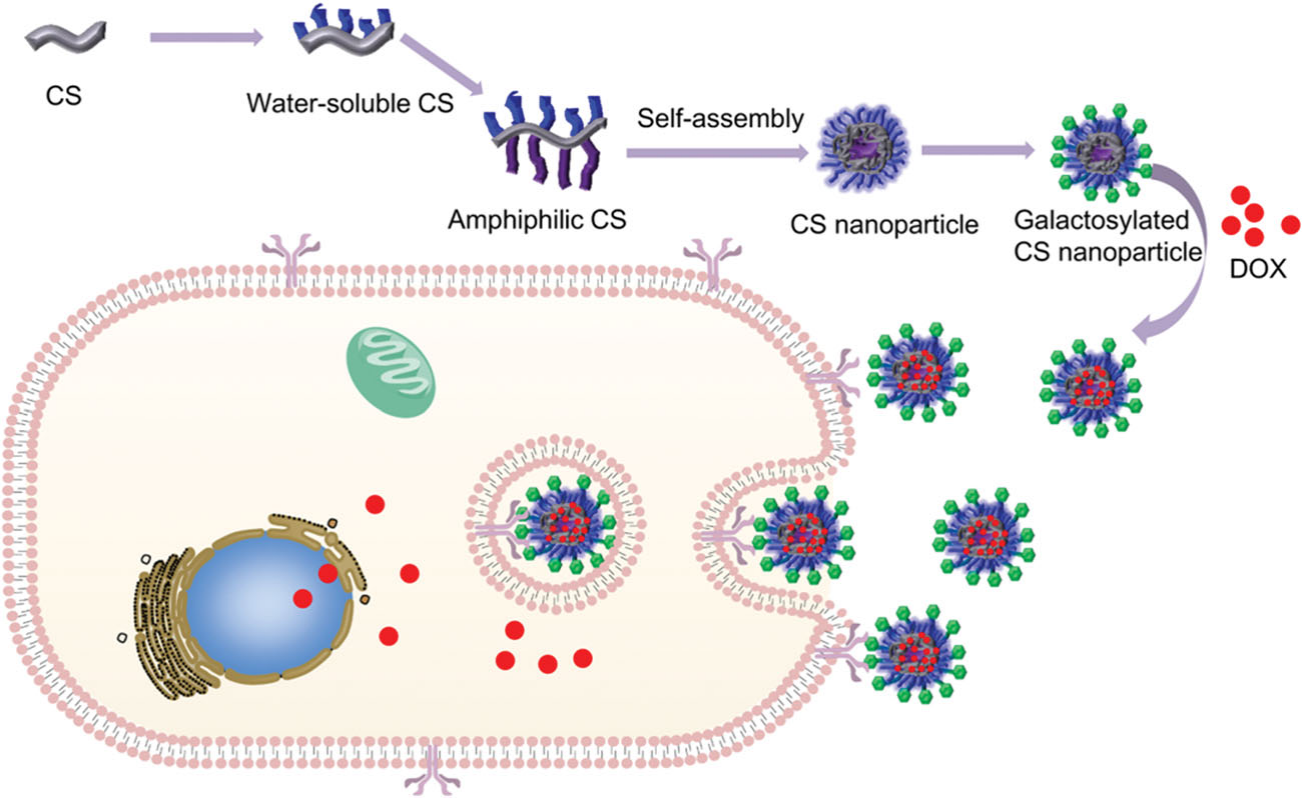
Schematic illustration of self-assembled chitosan nanoparticles and the mechanism of overcoming HCC via asialoglycoprotein receptor.

In this work, we aimed to develop several novel self-assembled chitosan nanoparticles to selectively overcome HCC via asialoglycoprotein receptor (see Scheme 1). The self-assembled chitosan nanoparticles were firstly synthesized by three steps: (1) Chitosan was modified with hydrophilic group (glycidol, Gly) to form water-soluble Gly-CS. (2) Gly-CS was modified with hydrophobic group (deoxycholic acid, DCA or vitamin E succinate, VE) to form amphiphilic chitosan, which could form self-assembled nanoparticles in aqueous solution. Apart from serving as hydrophobic group, DCA could also promote the liver-targeting ability of nanoparticle by hepatocyte transporters; VE could promote the efficacy of anticancer drugs (Gagic et al., 2016; Xiao et al., 2019). (3) Lactobionic acid was grafted onto the self-assembled nanoparticles to form galactosylated chitosan nanoparticles, and anticancer drug doxorubicin (DOX) was loaded into the nanoparticles. The properties of nanoparticles were then characterized. Furthermore, the *in vitro* cellular uptake, cell viability, wound healing experiments, and *in vivo* anticancer efficacy and toxicity of nanoparticles were performed.

## 2. Materials and methods

### 2.1. Materials

Chitosan (Mw 50 kDa, degree of deacetylation 80%) was purchased from Golden-Shell Pharmaceutical Co. Ltd. (Zhengjiang, China). Doxorubicin hydrochloride (DOX·HCl) was obtained from Meilun Biotech Co., Ltd. (Dalian, China). 1-(3-Dimethylaminopropyl)-3-ethylcarbodiimide hydrochloride (EDC·HCl), N-hydroxysuccinimide (NHS), acetic acid, glycidol, vitamin E succinate (VE), deoxycholic acid (DCA), lactobionic acid, D_2_O, and CD_3_COOD were supplied by 9 Ding Chemistry (Shanghai, China). KBr was bought from Adamas-Beta Co., Ltd. (Adamas-Beta, Shanghai, China). 96-well plates (In vitro scientific) were obtained from Xinyou Biotechnology Co., Ltd. (Hangzhou, China). Syringe-driven filters were supported by Jet Bio-Filtration Co., Ltd. (Guangzhou, China). Dulbecco’s modified Eagle’s medium (DMEM), RPMI 1640 medium, trypsin, penicillin, streptomycin, 3-(4,5-dimethylthiazol-2-yl)-2,5-diphenyltetrazolium bromide (MTT) and Hoechst33342 were bought from Solarbio Science & Technology Co., Ltd. (Beijing, China). Fetal bovine serum (FBS) was purchased from Zhejiang Tianhang Sijiqing Biotechnology Co., Ltd., China. Alanine aminotransferase (ALT) kit, aspartate aminotransferase (AST) kit, lactate dehydrogenase (LDH) kit, blood urea nitrogen (BUN) kit, and creatine kinase (CK) kit were purchased from JianCheng Bioengineering Institute (NanJing, China). Male BALB/c mice (18-22 g) were purchased from Liaoning Changsheng Biotechnology (Liaoning, China). All animal experiments were performed according to the Guiding Principles for Care and Use Committee of Experiment Animal in Dalian Medical University. All of the chemicals were analytical grade and used without further purification.

### 2.2. Synthesis of glycidol-chitosan (Gly-CS)

**Figure 1.**
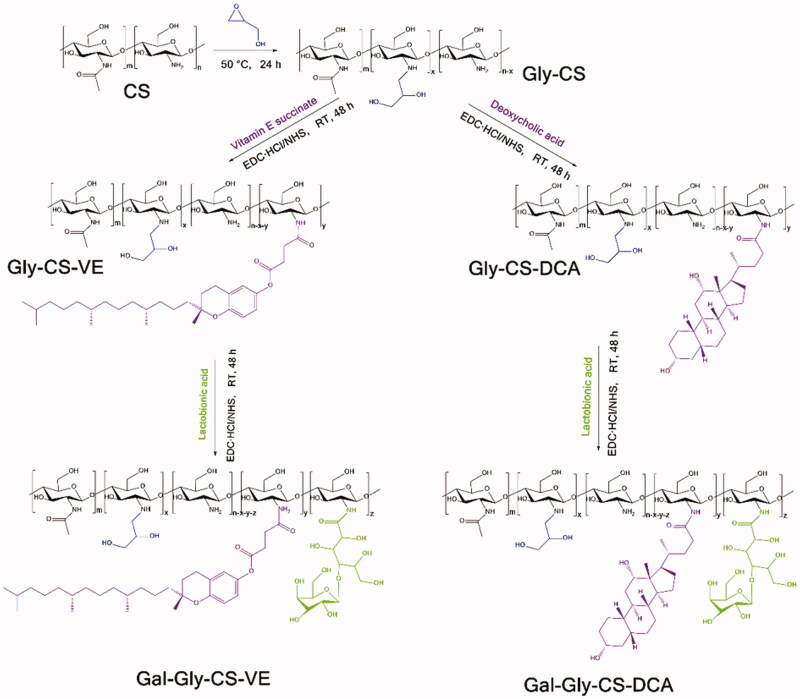
Synthetic scheme of chitosan derivatives (Gly-CS, Gly-CS-VE, Gal-Gly-CS-VE, Gly-CS-DCA, and Gal-Gly-CS-DCA).

As illustrated in Figure 1, glycidol-chitosan (Gly-CS) was synthesized according to previously reported method with some modifications (Zhou et al., 2010; Zhang & Cao, 2014). Briefly, 1 g chitosan (containing 4.95 mmol glucosamine residue) was dissolved in 100 mL acetic acid solution (1%, v/v), and filtered by 0.22 μm membrane filter. Then, 657 μL glycidol (9.9 mmol, twice of glucosamine residue) was added dropwise to chitosan solution, and reacted for 24 h at 50 °C. The reaction mixture was dialyzed with a dialysis bag (MWCO 3500 Da) on a magnetic stirrer against Milli-Q deionized water, which was replaced with fresh water every 4 h. The sample was dialyzed for 72 h to ensure the unreacted glycidol was completely removed. At last, it was lyophilized by a freeze-dryer (SCIENTZ-10N, Ningbo Scientz Biotechnology Co., Ltd., Zhejiang, China) to obtain the Gly-CS with the yield of 91.6%.

### 2.3. Synthesis of amphiphilic chitosan (Gly-CS-VE, Gly-CS-DCA)

As illustrated in Figure 1, 500 mg Gly-CS (containing 2.48 mmol glucosamine residue) was dissolved in 50 mL Milli-Q deionized water. 66 mg VE (0.124 mmol, chitosan glucosamine residue/VE mole ratio of 100:5), 23 mg EDC·HCl (0.124 mmol), and 14 mg NHS (0.124 mmol) were dissolved in 25 mL DMSO, and stirred for 30 min to activate VE. Subsequently, the two kinds of solutions were mixed at room temperature, and reacted for 24 h. Then, they were purified by a dialysis bag (MWCO 3500 Da) on a magnetic stirrer for 72 h to remove the unreacted materials. Finally, the solution was filtered by 0.22 μm membrane filter to remove large particles. Gly-CS-VE was obtained by lyophilization with the yield of 63.2% and stored at 4 °C for further use. Furthermore, chitosan was also reacted with VE under the chitosan glucosamine residue/VE mole ratio of 100:1.25, 100:2.5 or 100:10, and the Gly-CS-VE was obtained for further use.

For synthesis of Gly-CS-DCA, the similar procedure was carried out. 500 mg Gly-CS reacted with 292 mg DCA (0.744 mmol, chitosan glucosamine residue/VE mole ratio of 100:30), 198 mg EDC·HCl (0.744 mmol) and 120 mg NHS (0.744 mmol) for 24 h. Then, they were purified by dialysis and filtered by 0.22 μm membrane filter. The Gly-CS-DCA was obtained by lyophilization with the yield of 71.0%. Furthermore, chitosan was also reacted with DCA under the chitosan glucosamine residue/DCA mole ratio of 100:10, 100:20, or 100:40, and the Gly-CS-DCA was obtained for further use.

### 2.4. Synthesis of galactosylated chitosan nanoparticles (Gal-Gly-CS-VE, Gal-Gly-CS-DCA)

As illustrated in Figure 1, 171 mg Gal-Gly-CS-VE (or Gal-Gly-CS-DCA, containing 1.06 mmol glucosamine residue), 454 mg lactobionic acid (1.27 mmol, chitosan glucosamine residue/galactose residue mole ratio of 100:120), 849 mg EDC·HCl (4.42 mmol), and 510 mg NHS (4.42 mmol) were dissolved in 75 mL Milli-Q deionized water. The mixture was reacted for 24 h at room temperature, and dialyzed by a dialysis bag (MWCO 3500 Da). Three days later, the unreacted materials were completely removed and the solution was filtered by 0.22 μm membrane filter. Finally, Gal-Gly-CS-VE (or Gal-Gly-CS-DCA) was obtained by lyophilization and stored at 4 °C for further use. The yields of Gal-Gly-CS-VE and Gal-Gly-CS-DCA were 55.0% and 60.0%, respectively. Furthermore, chitosan nanoparticles were also reacted with lactobionic acid under the chitosan glucosamine residue/galactose residue mole ratio of 100:60, 100:90, or 100:150, and the Gal-Gly-CS-VE (or Gal-Gly-CS-DCA) was obtained for further use.

### 2.5. Preparation of DOX loaded chitosan nanoparticles

The loading of DOX was carried out by dialysis method (Zhou et al., 2010). Briefly, DOX·HCl was dissolved in DMSO with the concentration of 1 mg/mL, and 50 μL triethylamine was added to neutralize DOX·HCl for 12 h. Subsequently, the obtained DOX base was respectively mixed with the nanodispersions of Gly-CS-VE, Gly-CS-DCA, Gal-Gly-CS-VE, or Gal-Gly-CS-DCA, where the mass ratio of chitosan nanoparticles/DOX·HCl was 5: 1. The mixtures were stirred for 24 h and dialyzed against Milli-Q deionized water for 72 h (MWCO 3500 Da). They were finally lyophilized to obtain the DOX loaded chitosan nanoparticles.

To determine the encapsulation efficiency (EE) and drug loading (DL) of DOX, a certain amount of DOX-loaded chitosan nanoparticles (Gly-CS-VE, Gly-CS-DCA, Gal-Gly-CS-VE, or Gal-Gly-CS-DCA) was dispersed in deionized water. The concentration of DOX was measured by a UV-Vis spectrophotometer (UV759CRT, Yoke, Shanghai, China) at 480 nm. The EE and DL were calculated by the following equations:

(1) $$\mathrm{EE}(\%)=\frac{\text{Amount of loaded DOX}}{\text{Total amount of DOX}}\times 100$$

(2) $$\mathrm{DL}(\%)=\frac{\text{Amount of loaded DOX}}{\text{Total amount of chitosan nanoparticles}}\times 100$$

### 2.6. Characterizations

^1^H NMR spectra of CS, Gly-CS, Gly-CS-VE, Gly-CS-DCA, Gal-Gly-CS-VE, and Gal-Gly-CS-DCA were recorded by a Varian Mercury Plus 400 MHz NMR spectrometer (Varian, Palo Alto, CA, USA). The solvent was D_2_O with 1% (v/v) CD_3_COOD.

Fourier transform infrared (FT-IR) spectra of CS, Gly-CS, Gly-CS-VE, Gly-CS-DCA, Gal-Gly-CS-VE, and Gal-Gly-CS-DCA were recorded by an IRAffinity-1 FT-IR spectrophotometer (Shimadzu, Kyoto, Japan). Each sample was compressed with KBr powder into a pellet and scanned from 4000 to 400 cm^−1^, with a resolution of 4 cm^−1^.

The morphologies of Gly-CS-VE, Gly-CS-DCA, Gal-Gly-CS-VE, and Gal-Gly-CS-DCA nanoparticles were observed by a transmission electron microscopy (TEM) (JEM-2000EX, JEOL, Kyoto, Japan). The nanodispersion of nanoparticles was respectively dropped on a carbon-coated copper grid, and negatively strained with 2% phosphotungstic acid for 6–8 min. The grid was dried by filter paper and observed at an acceleration voltage of 120 kV.

The average particle sizes, zeta potentials, and polydispersity indexes of Gly-CS-VE, Gly-CS-DCA, Gal-Gly-CS-VE, and Gal-Gly-CS-DCA nanoparticles were measured by dynamic light scattering particle size analyzer (Nano ZS90, Malvern, Worcestershire, UK).

### 2.7. *In vitro* drug release assay

The *in vitro* release of DOX from Gly-CS-VE, Gly-CS-DCA, Gal-Gly-CS-VE, and Gal-Gly-CS-DCA nanoparticles was determined by dialysis method. Briefly, 2 mL Gly-CS-VE, Gly-CS-DCA, Gal-Gly-CS-VE, or Gal-Gly-CS-DCA nanodispersion containing 150 μg/mL DOX was added in a dialysis bag (MWCO 3500 Da) and then immersed in 15 mL phosphate buffer solution (pH 7 or pH 5, containing 0.5% Tween 80) within a 20 mL centrifuge tube. The tube was placed in an incubator shaker, shaking at 37 °C with a speed of 100 rpm. At predetermined time points, 5 mL release medium was taken out, and equal volume of fresh medium was added. The concentration of DOX in each sample was measured by a UV-Vis spectrophotometer at 480 nm. The cumulative release of DOX was calculated. Three parallel samples for each group were measured.

### 2.8. Cell culture

Human HCC HepG2 cells were cultured in DMEM medium supplemented with 10% (v/v) FBS, 100 U/mL penicillin, and 100 U/mL streptomycin at 37 °C in a humidified incubator containing 5% CO_2_.

Murine HCC H22 cells were cultured in RPMI 1640 medium supplemented with 10% (v/v) FBS, 100 U/mL penicillin, and 100 U/mL streptomycin at 37 °C in a humidified incubator containing 5% CO_2_.

### 2.9. *In vitro* cellular uptake assay

HepG2 cells were seeded on 12-well plates at an initial density of 1 × 10^5^ cells/well and cultured overnight. Then, the cells were treated with free DOX, DOX-loaded Gly-CS-VE, Gly-CS-DCA, Gal-Gly-CS-VE, or Gal-Gly-CS-DCA nanoparticles (the final concentration of DOX was 5 μg/mL). After 2 h or 4 h incubation, the cells were washed with PBS and stained with Hoechst33342 for 15 min. Subsequently, the cells were washed with PBS three times and observed under a fluorescence microscope (IX81, Olympus, Tokyo, Japan).

To further quantify the uptake of DOX, HepG2 cells were seeded on 6-well plates (1 × 10^6^ cells/well) and cultured overnight. Then, they were incubated with free DOX, DOX-loaded Gly-CS-VE, Gly-CS-DCA, Gal-Gly-CS-VE, and Gal-Gly-CS-DCA nanoparticles (the final concentration of DOX was 5 μg/mL). After 2 or 4 h incubation, the cells were washed with PBS three times, harvested by trypsin digestion, and suspended in PBS. The collected cells were filtered using nylon mesh and measured by a flow cytometer (Becton Dickinson, San Jose, USA).

### 2.10. *In vitro* cytotoxicity assay

The cytotoxicity of blank chitosan nanoparticles and DOX-loaded chitosan nanoparticles was evaluated by MTT method. HepG2 cells were seeded at a density of 5 × 10^3^ cells/well in 96-well plates and cultured overnight. The medium was then replaced with 100 μL fresh medium containing blank chitosan nanoparticles or DOX-loaded chitosan nanoparticles (Gly-CS-VE, Gly-CS-DCA, Gal-Gly-CS-VE, and Gal-Gly-CS-DCA) with different concentrations. After 48 h of incubation, the cells were washed with PBS three times, and incubated for another 4 h with fresh medium containing 20 μL MTT (5 mg/mL). Then, the medium was carefully taken away and 200 μL DMSO was added to dissolve the formazan crystals. The absorbance of each well was tested at 570 nm by a microplate reader (Multiskan Ascent 354, Thermo Fisher Scientific, Rockford, IL, USA). Cells without any sample were incubated as parallel negative controls, and the wells with added culture medium without cells served as blank controls for zero absorbance at the same time. The cell viability was calculated as the following equation:

(3) $$\text{Cell}\;\text{vability}\;(\%)=\frac{\text{Abs}(\text{sample})-\text{Abs}(\text{blank})}{\text{Abs}(\text{control})-\text{Abs}(\text{blank})}\times 100$$

### 2.11. *In vitro* wound healing assay

The cell migration was evaluated by wound healing assay. In brief, HepG2 cells were seeded on 6-well plates at a density of 1 × 10^5^ cell/well and incubated to reach full confluence. The monolayer cells were scratched by a sterile 10 μL pipette tip and the medium was replaced by fresh DMEM containing 20% FBS. Subsequently, the cells were treated by free DOX, DOX-loaded Gly-CS-VE, Gly-CS-DCA, Gal-Gly-CS-VE, and Gal-Gly-CS-DCA nanoparticles (the final concentration of DOX was 5 μg/mL). After incubated for 0, 24, and 48 h, the images were taken by an inverted microscope. The distance of each group was analyzed by Image-Pro-Plus software (Media Cybernetics, Silver Spring, USA). The wound healing rate (%) was calculated by the following equation:

(4) $$\text{Wound}\;\text{healing}\;\text{rate}\;(\%)=100-\frac{\text{distance}\;\text{of}\;\text{scratched}\;\text{area}\;\text{at}\;24h\;\text{or}\;48h}{\text{distance}\;\text{of}\;\text{scratched}\;\text{area}\;\text{at}\;0h\;}\times 100$$

### 2.12. *In vivo* anticancer efficacy study

The *in vivo* anticancer efficacy was evaluated by the H22 tumor xenograft model with some modifications (Qi et al., 2015). Male BALB/c mice (18–22 g) were purchased from Liaoning Changsheng Biotechnology. The animals were housed in cages under standard 12 h light/dark cycle condition at room temperature with free access to food and water in a pathogen-free environment. After acclimated for a week, the mice were subcutaneously injected with H22 cells (1 × 10^7^ cells in 200 μL PBS) at the right axilla. When the tumor volume reached 100–150 mm^3^, the mice were randomly divided into six groups (*n* = 6). They were intravenously injected via the tail vein with (I) saline, (II) free DOX, (III) Gly-CS-VE, (IV) Gal-Gly-CS-VE, (V) Gly-CS-DCA, and (VI) Gal-Gly-CS-DCA every 3 days. The samples were prepared by dissolving DOX·HCl or dispersing DOX-loaded chitosan nanoparticles in PBS with the DOX concentration of 500 μg/mL. The dosage of injected DOX was maintained at 5 mg/kg. The tumor volume and body weight were recorded every day. The tumor volume was calculated using the following equation:

(5) $$\text{Tumor volume }(V)=a\times b^{2}/2$$

where a was the longest length and b was the shortest length of the tumors.

After 11 days of treatment, all mice were euthanized to excise tumors. The tumors were washed by saline, dried by filter paper, weighed, and imaged. Finally, they were fixed in 4% paraformaldehyde and then imbedded in paraffin block for histologic section. Hematoxylin and eosin (H&E) staining was performed to evaluate the pathological changes.

### 2.13. *In vivo* toxicity study

After 11 days of treatment, all mice were euthanized with the blood samples and major organs (heart, liver, spleen, lung, and kidney) collected. The blood was centrifuged at 3000 rpm for 5 min to obtain plasma. ALT, AST, LDH, BUN, and CK were measured by kits according to the instructions. Major organs including heart, liver, spleen, lung, and kidney were fixed in 4% paraformaldehyde and then imbedded in paraffin block for histologic section. Hematoxylin and eosin (H&E) staining were performed to evaluate the pathological changes.

### 2.14. Statistical analysis

All data presented in this work were expressed as means ± SD. Statistical differences between different groups were analyzed with Student’s *t*-test and one-way analysis of variance. Differences with *p* < 0.05(*), *p* < 0.01(**), and *p* < 0.001 (***) were considered statistically significant.

## 3. Results and discussion

### 3.1. Characterizations of chitosan derivatives and nanoparticles

The synthetic route of chitosan derivatives (Gly-CS, Gly-CS-VE, Gal-Gly-CS-VE, Gly-CS-DCA, and Gal-Gly-CS-DCA) was shown in Figure 1. CS was firstly reacted with hydrophilic glycidol via nucleophilic ring-opening reaction under the catalysis of hydrogen ions (Pan et al., 2013). The formed Gly-CS was water-soluble and it was subsequently reacted with hydrophobic vitamin E succinate (VE) or deoxycholic acid (DCA) via EDC/NHS mediated amidation. The amphiphilic Gly-CS-VE or Gly-CS-DCA was then reacted with lactobionic acid to obtain galactosylated chitosan derivatives Gal-Gly-CS-VE or Gal-Gly-CS-DCA.

**Figure 2.**
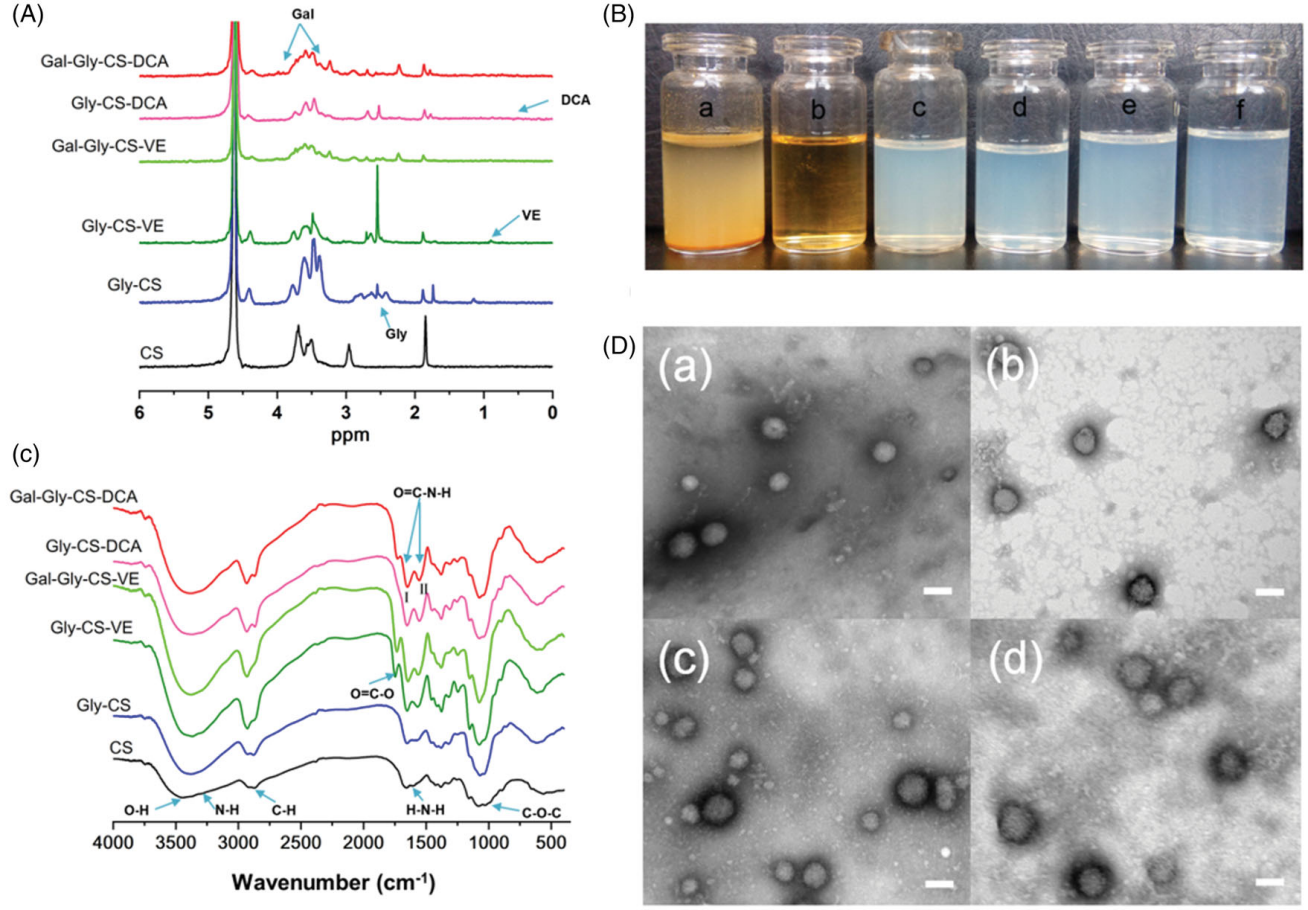
Characterizations of chitosan derivatives and nanoparticles. (A) FT-IR spectra of chitosan derivatives. (B) ^1^H NMR spectra of chitosan derivatives. (C) Appearance of aqueous solutions: (a) CS, (b) Gly-CS, (c) Gly-CS-VE, (d) Gal-Gly-CS-VE, (e) Gly-CS-DCA, (f) Gal-Gly-CS-DCA. (D) TEM images of chitosan nanoparticles: (a) Gly-CS-VE, (b) Gal-Gly-CS-VE, (c) Gly-CS-DCA, (d) Gal-Gly-CS-DCA, scale bar = 100 nm.

The characterizations of chitosan derivatives and nanoparticles were shown in Figure 2 and Table 1. By comparing the ^1^H NMR spectra of CS and Gly-CS (Figure 2(A)), the peaks in the region of 2.4–2.9 ppm represented the proton of -N-CH_2_- group in glycidol moiety, while the peak at 4.4 ppm represented the proton of –OH group in glycidol moiety. In the spectrum of Gly-CS-VE, the peak at 0.9 ppm was attributed to the proton of methyl group in vitamin E structure. Similarly, the weak signal at 0.77 ppm in the spectrum of Gly-CS-DCA was affiliated to the proton of deoxycholic acid fragment. For Gal-Gly-CS-VE and Gal-Gly-CS-DCA, the signal at 4.35 ppm was attributed to methyl proton of galactose moiety (Wang et al., 2018). The newly appeared peaks at 3.3–3.8 ppm were ascribed to the ring proton of galactose moiety, which was partly overlapped with the signal of chitosan backbone (Guo et al., 2013; Zhang & Cao, 2014). As the liver-targeting ligands, the amounts of galactose residues could affect the liver-targeting effect. Hence, we subsequently calculated the substitution degrees (DS) of galactose residues in Gal-Gly-CS-VE and Gal-Gly-CS-DCA by ^1^H NMR according to the following equation (Wang et al., 2010):

(6) $${DS}_{\mathrm{Gal}}=\frac{H_{\mathrm{Gal}-\mathrm{Gly}-CS-VE/\mathrm{DCA}(\delta 3.25-4.0)}-H_{\mathrm{Gly}-CS-VE/\mathrm{DCA}(\delta 3.25-4.0)}}{12}\times 100\%$$

**Table 1.** Characterizations of blank chitosan nanoparticles and DOX-loaded chitosan nanoparticles: particle size, zeta potential, PDI, EE, and DL.

	Blank chitosan nanoparticles			DOX-loaded chitosan nanoparticles				
	Size (nm)	Zeta potential (mV)	PDI	Size (nm)	Zeta potential (mV)	PDI	EE %	DL %
Gly-CS-VE	178.90 ± 2.30	19.80 ± 0.44	0.147 ± 0.003	198.57 ± 2.46	52.77 ± 0.49	0.156 ± 0.024	56.09 ± 0.10	10.08 ± 0.02
Gal-CS-VE	142.07 ± 4.89	7.57 ± 0.45	0.176 ± 0.015	165.10 ± 3.57	22.57 ± 3.43	0.196 ± 0.033	57.82 ± 0.04	10.36 ± 0.01
Gly-CS-DCA	142.73 ± 1.10	24.47 ± 0.97	0.164 ± 0.017	165.07 ± 1.46	40.00 ± 1.20	0.139 ± 0.021	58.91 ± 0.14	10.51 ± 0.02
Gal-Gly-CS-DCA	159.90 ± 2.60	17.17 ± 1.55	0.134 ± 0.018	156.53 ± 2.78	30.20 ± 5.48	0.164 ± 0.015	54.21 ± 0.26	9.64 ± 0.04

where H_Gal-Gly-CS-VE/DCA(δ3.25-4.0)_ was the amount of hydrogen atoms of Gal-Gly-CS-VE or Gal-Gly-CS-DCA at δ 3.25–4.0 ppm. H_Gly-CS-VE/DCA(δ3.25-4.0)_ was the amount of hydrogen atoms of Gly-CS-VE or Gly-CS-DCA at δ 3.25–4.0 ppm. The results should that the substitution degrees (DS) of galactose residues were 34.66% in Gal-Gly-CS-VE and 33.75% in Gal-Gly-CS-DCA.

The structures of chitosan derivatives were also confirmed by FT-IR (shown in Figure 2(B)). In the spectrum of CS, some major groups could be identified. The broad band of 3100–3700 cm^−1^ belonged to the O–H and N–H stretching vibration. The band at 2900 cm^−1^ was attributed to the C-H stretching vibration, while the band at 1079 cm^−1^ indicated the C–O–C stretching vibration (Barbosa et al., 2019). The band at 1594 cm^−1^ represented the N–H bending vibration of primary amine. As the primary amine was the reactive group to prepare the CS chitosan derivatives, the band of primary amine was gradually disappeared in the spectra of Gly-CS, Gly-CS-VE, Gal-Gly-CS-VE, Gly-CS-DCA, and Gal-Gly-CS-DCA. Correspondingly, the signal of amide linkage was enhanced at the bands of 1655 cm^−1^ (amide I band, C=O stretching) and 1553 cm^−1^ (amide II band, N–H bending and stretching). For Gly-CS-VE, the new band at 1747 cm^−1^ was attributed to the C=O stretching vibration of ester linkage. The results of FT-IR and ^1^H NMR demonstrated the functional groups were successfully grafted on the chitosan backbone. Furthermore, the appearances of CS, Gly-CS, Gly-CS-VE, Gal-Gly-CS-VE, Gly-CS-DCA, and Gal-Gly-CS-DCA in aqueous solutions were observed. As shown in Figure 2(C), CS was insoluble in deionized water and precipitated on the bottom. However, Gly-CS could be thoroughly dissolved in water and formed a clear and transparent solution. This was because the glycidol moiety could significantly improve the hydrophilcity of CS. For Gly-CS-VE, Gly-CS-DCA, Gal-Gly-CS-VE, and Gly-CS-DCA, their nanodispersions were light blue opalescent with obvious Tyndall effect, indicating they formed self-assembled chitosan nanoparticles in water. The nanoparticles were quite stable in solution, with no precipitate observed for several months. In addition, the TEM morphologies of Gly-CS-VE, Gly-CS-DCA, Gal-Gly-CS-VE, and Gal-Gly-CS-DCA nanoparticles were exhibited in Figure 2(D). All four kinds of nanoparticles were spherical in shape, uniform, and well-dispersed, with the particle sizes of approximately 100 nm. Furthermore, their average particle sizes, zeta potentials and polydispersity indexes measured by dynamic light scattering (DLS) were also listed in Table 1. The average particle sizes of Gly-CS-VE, Gly-CS-DCA, Gal-Gly-CS-VE, and Gal-Gly-CS-DCA were 178.90 ± 2.30 nm, 142.07 ± 4.89, 142.73 ± 1.10, and 159.90 ± 2.60 nm, respectively. We found the particles sizes obtained from DLS were larger than that of TEM. It was because the nanoparticles had hydration shells in aqueous solution during DLS measurement, while they were dried and shrinked during TEM measurement. The surface charges of four nanoparticles were positive, with the zeta potentials of 19.80 ± 0.44, 7.57 ± 0.45, 24.47 ± 0.97, and 17.17 ± 1.55 mV. The PDI of four nanoparticles was less than 0.2, meaning they were homogeneous. The nanometer scale size of Gly-CS-VE, Gly-CS-DCA, Gal-Gly-CS-VE, and Gal-Gly-CS-DCA could be beneficial for tumor targeting via the EPR effect, and their positive charge could further improve the endocytosis by cancer cells. It is worth to note that the effects of VE, DCA, and lactobionic acid on the properties of chitosan nanoparticles were also studied by comparing their different mole ratios to chitosan glucosamine residue. The nanoparticles mentioned above were selected as the optimal prescription, please see the Supplemental material.

### 3.2. Characterizations of DOX-loaded chitosan nanoparticles

**Figure 3.**
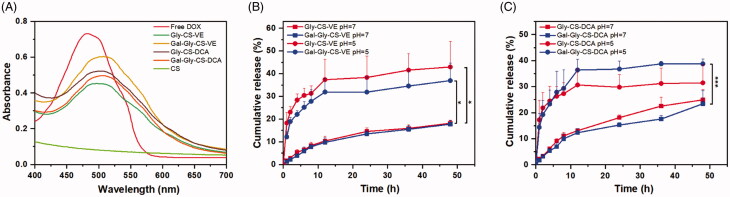
(A) UV-Vis absorption spectra of DOX solution, CS and DOX loaded chitosan nanoparticles. (B) *In vitro* release of DOX from Gly-CS-VE and Gal-Gly-CS-VE nanoparticles at pH 5 and 7. (C) *In vitro* release of DOX from Gly-CS-DCA and Gal-Gly-CS-DCA nanoparticles at pH 5 and 7, *p < 0.05, ***p < 0.001.

Anticancer drug DOX was loaded in the four chitosan nanoparticles by dialysis method. The UV-Vis absorption spectra of DOX solution, CS, and DOX-loaded chitosan nanoparticles were shown in Figure 3(A). The maximum absorption wavelength of free DOX solution was 480 nm, while no intense peak was found in CS solution within 400–700 nm. For DOX loaded Gly-CS-VE, Gal-Gly-CS-VE, Gly-CS-DCA, and Gal-Gly-CS-DCA nanodispersions, they displayed similar absorption spectra to DOX solution with a 20 nm redshift. This indicated DOX was successfully loaded in chitosan nanoparticles, and it can be quantified by UV-Vis absorption spectra. The characterizations of DOX-loaded chitosan nanoparticles were shown in Table 1. The average particle sizes of DOX loaded Gly-CS-VE, Gal-CS-VE, Gly-CS-DCA, and Gal-Gly-CS-DCA nanoparticles were 198.57 ± 2.46, 165.10 ± 3.57, 165.07 ± 1.46, and 156.53 ± 2.78 nm, which were slightly larger than the blank chitosan nanoparticles due to the payload of cargoes. The zeta potentials of four DOX loaded chitosan nanoparticles were 52.77 ± 0.49, 22.57 ± 3.43, 40.00 ± 1.20 and 30.20 ± 5.48 mV, which were more positive than blank chitosan nanoparticles. This was because DOX contained an amino group, and it could increase the positive charges of nanoparticles by adsorbing on their surfaces. The encapsulation efficiencies of four DOX-loaded chitosan nanoparticles were in the range of 54–59%, and the drug loadings were in the range of 9.5–10.5%.

### 3.3. *In vitro* drug release

The release of DOX from chitosan nanoparticles at different pH conditions was measured by dialysis method. As shown in Figure 3(B), the release of DOX from Gly-CS-VE and Gal-Gly-CS-VE nanoparticles exhibited sustained release manners at pH 7, with only 15% of DOX diffused out in 48 h. However, the release rates were significantly enhanced at pH 5, with the cumulative release reached to about 40% in 48 h. In Figure 3(C), the release of DOX from Gly-CS-DCA and Gal-Gly-CS-DCA nanoparticles showed a similar manner. The faster release speed of DOX in acidic condition could be attributed to the protonation of amino group in DOX base. The pH-dependent release manner was beneficial for DOX accumulation in tumor tissue, as the tumor microenvironment was acidic (pH 4–5 in lysosomes and pH 5–6 in endosomes).

### 3.4. *In vitro* cellular uptake

**Figure 4.**
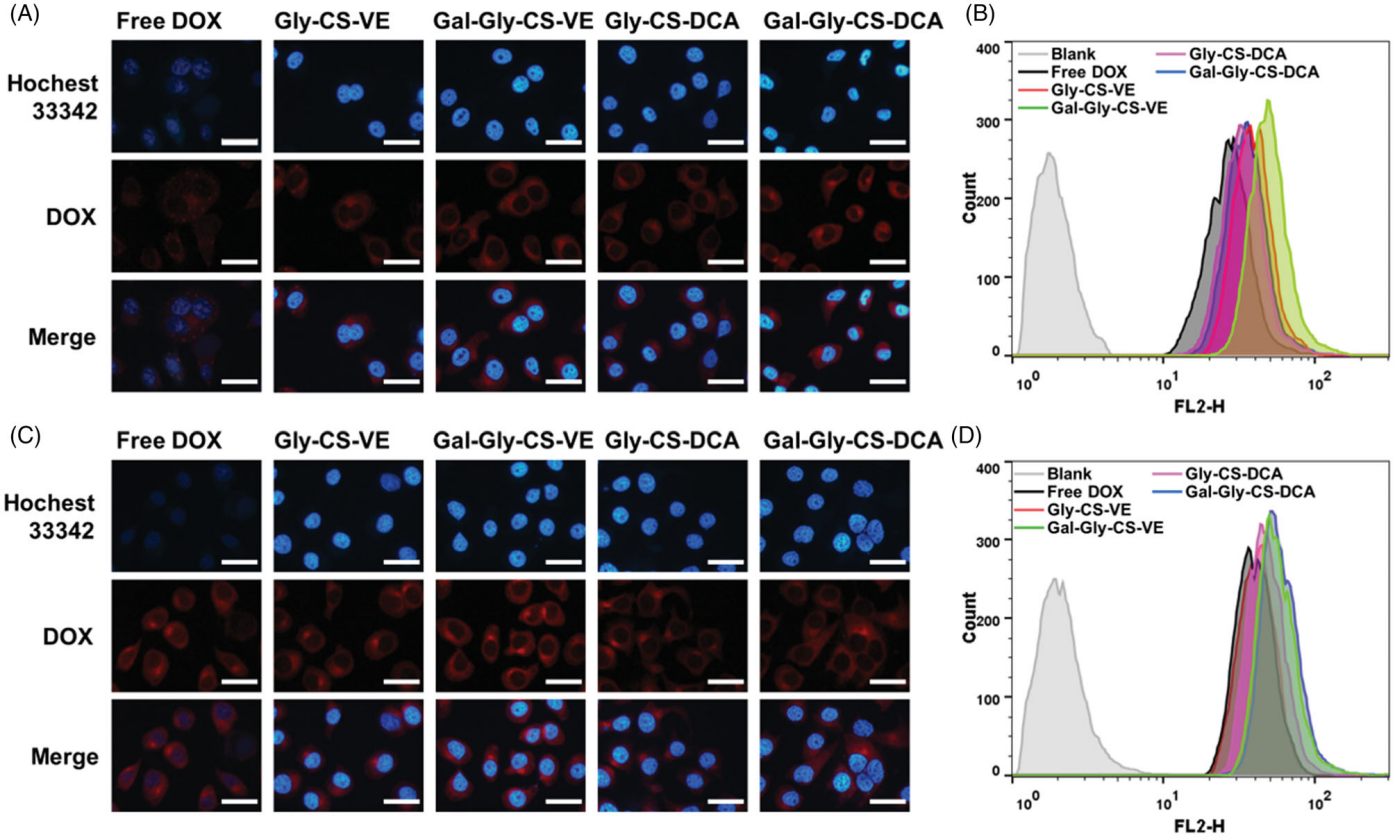
Cellular uptake of DOX and DOX loaded chitosan nanoparticles by HepG2 cells. (A) Fluorescence microscope images and (B) flow cytometer analysis after 2 h incubation. (C) Fluorescence microscope images and (D) flow cytometer analysis after 4 h incubation. Scale bar = 20 μm.

Cellular uptake is a key step for anticancer drugs to reach the pharmacologic target. In this work, HepG2 cells were used to evaluate the cellular uptake, as ASGPR were overexpressed on HepG2 cells. Free DOX and DOX loaded Gly-CS-VE, Gal-Gly-CS-VE, Gly-CS-DCA, and Gal-Gly-CS-DCA nanoparticles were incubated with HepG2 cells for 2 or 4 h, and the internalization was analyzed by fluorescence microscope. As seen in Figure 4(A,C), the blue fluorescence represented the signal of Hochest 33342 excited by 405 nm laser, which stained the cell nuclei. The red fluorescence corresponded to the signal of DOX excited by 488 nm laser, and the intensity could reflect the amount of internalized DOX. It was clear that free DOX and DOX-loaded nanoparticles were uptaken in a time-dependent way, as the red fluorescence intensities were increasing with time extension. In addition, the four DOX-loaded nanoparticles groups all exhibited stronger red fluorescence intensities than the free DOX group. This indicated chitosan nanoparticles could enhance the cellular uptake of DOX by HepG2 cells, which could be attributed to the positive charge of chitosan. Moreover, we found the galactosylated Gal-Gly-CS-VE and Gal-Gly-CS-DCA groups exhibited stronger red fluorescence intensities than the corresponding Gly-CS-VE and Gly-CS-DCA groups. To confirm the results of fluorescence microscope, the uptake of free DOX and DOX loaded nanoparticles was quantitatively evaluated by flow cytometer. The fluorescence of DOX was monitored in the FL2-H channel. As shown in Figure 4(B,D), HepG2 cells treated with PBS were set as blank groups and the fluorescence signals were near zero. Nevertheless, the cells treated by free DOX and DOX-loaded nanoparticles all exhibited fluorescence signals, and the nanoparticles groups showed stronger signals than the free DOX group. The mean fluorescence intensities of Gly-CS-VE, Gal-Gly-CS-VE, Gly-CS-DCA, and Gal-Gly-CS-DCA groups were respectively 1.03-fold, 1.45-fold, 1.20-fold, and 1.52-fold higher than that of free DOX group after 4 h incubation. The galactosylated Gal-Gly-CS-VE and Gal-Gly-CS-DCA nanoparticles showed higher internalization efficiency than Gly-CS-VE and Gly-CS-DCA nanoparticles, which could be ascribed to ASGPR-targeting effect of galactose group. The results of fluorescence microscope and flow cytometer both confirmed that chitosan nanoparticles could improve the uptake efficiency of DOX, and galactosylated Gal-Gly-CS-VE and Gal-Gly-CS-DCA nanoparticles could be more easily internalized by HepG2 cells via overexpressed ASGPR.

### 3.5. *In vitro* cytotoxicity

**Figure 5.**
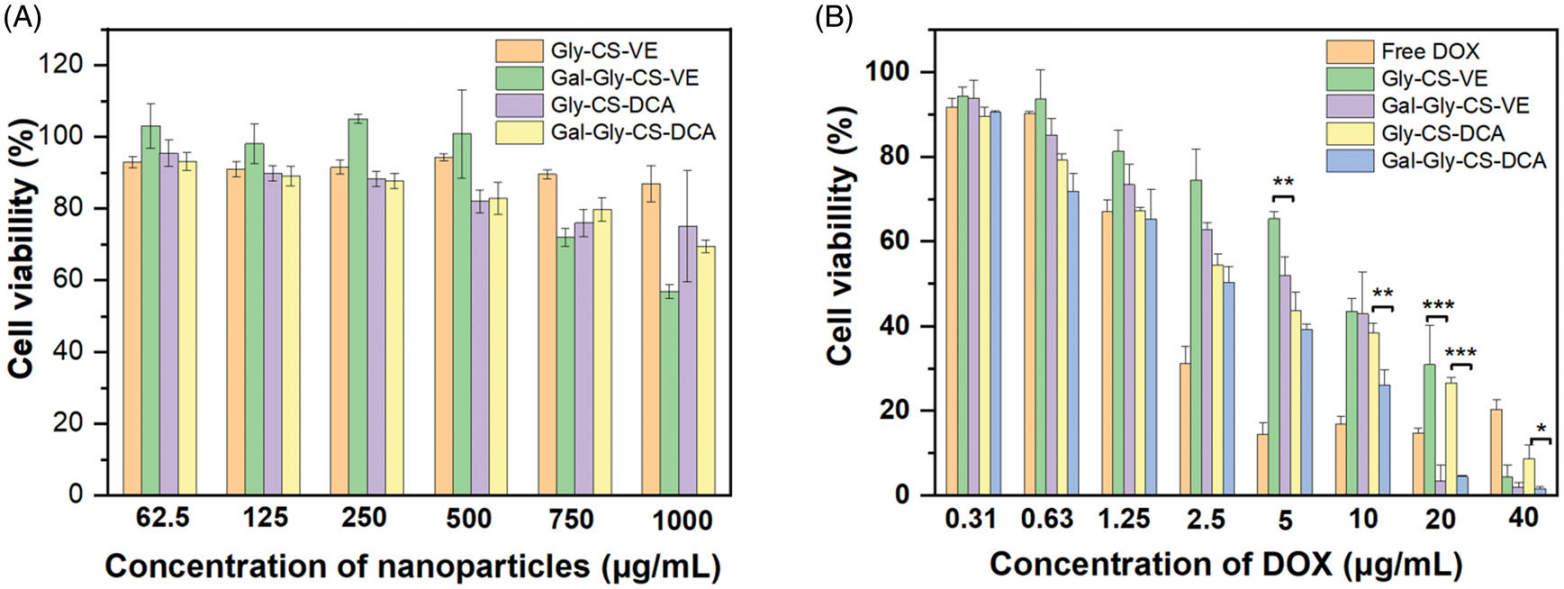
Cytotoxicity of blank chitosan nanoparticles (A) and DOX loaded chitosan nanoparticles (B) with different concentrations after incubated with HepG2 cells for 48 h, *p < 0.05, **p < 0.01, ***p < 0.001.

The *in vitro* cytotoxicity of blank chitosan nanoparticles and DOX loaded chitosan nanoparticles were evaluated by MTT method. Despite chitosan is considered as an excellent material with good biocompatibility and low toxicity, it is still toxic to cells at high dosage. Hence, the safety of blank chitosan nanoparticles was firstly evaluated. As seen in Figure 5(A), four kinds of chitosan nanoparticles Gly-CS-VE, Gal-Gly-CS-VE, Gly-CS-DCA, and Gal-Gly-CS-DCA all exhibited negligible cytotoxicities on HepG2 cells with the concentrations less than 750 μg/mL after 48 h incubation. Despite the Gal-Gly-CS-VE nanoparticles resulted in a cell viability of approximately 60% at the concentration of 1000 μg/mL, the cell viabilities of Gly-CS-VE, Gly-CS-DCA, and Gal-Gly-CS-DCA nanoparticles were all above 80%. In the subsequent experiments of measuring the cytotoxicity of DOX-loaded nanoparticles, the amounts of chitosan nanoparticles were less than 400 μg/mL. This assured the cytotoxicities were caused by DOX rather than the chitosan nanoparticles. The cytotoxicities of DOX and DOX-loaded chitosan nanoparticles were shown in Figure 5(B), with the concentrations of DOX ranging in 0.31–40 μg/mL. It was obvious that the cytotoxicities of DOX and DOX-loaded chitosan nanoparticles were concentration-dependent, as the cell killing efficiencies were gradually increased with higher DOX concentrations. Free DOX exhibited significant cell killing capability when the concentration exceeded 2.5 μg/mL. For DOX-loaded chitosan nanoparticles, it seemed they were less effective in killing HepG2 cells than free DOX, especially at the DOX concentrations of 2.5, 5, and 10 μg/mL. This was because DOX required a long time to release out of nanoparticles and got into the nucleus to damage DNA, after nanoparticles were internalized by HepG2 cells. Considering the release profiles of DOX (Figure 3), we found less than 40% of DOX could diffuse out of the nanoparticles within 48 h. This phenomenon was consistent with several previous reports (Lou et al., 2017; Sun et al., 2017). In spite of this, the four DOX-loaded chitosan nanoparticles all exhibited significantly higher cytotoxicity than free DOX at the concentration of 40 μg/mL. Furthermore, we found the cell killing capabilities of galactosylated Gal-Gly-CS-VE and Gal-Gly-CS-DCA nanoparticles were much higher than Gly-CS-VE and Gly-CS-DCA nanoparticles. This was in agreement with the enhanced cellular uptake efficiencies of galactosylated nanoparticles.

### 3.6. *In vitro* wound healing

**Figure 6.**
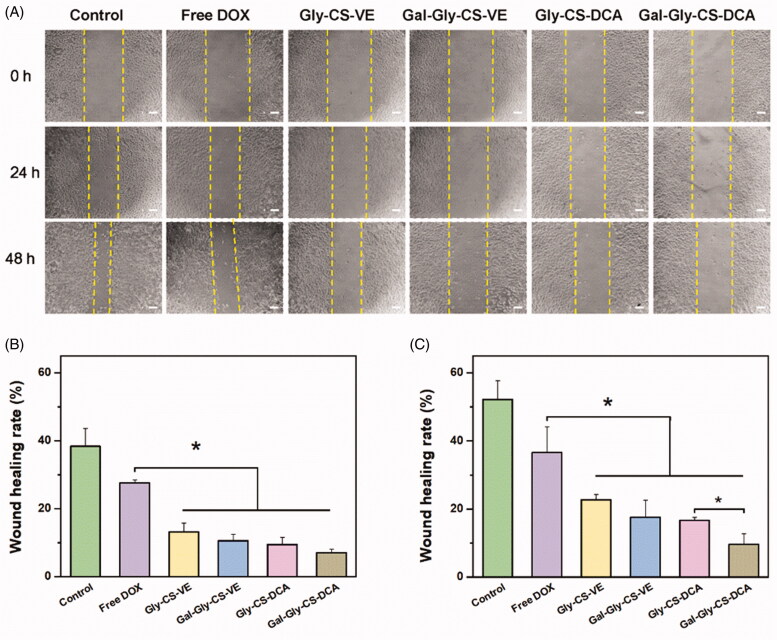
*In vitro* wound healing assay of DOX and DOX loaded chitosan nanoparticles on HepG2 cells. (A) Artificial scratch gaps of HepG2 cells after treated for 24 and 48 h. (B) The wound healing rates of each group after 24 h treatment. (C) The wound healing rates of each group after 48 h treatment, *p < 0.05.

The anti-cell migration effects of DOX-loaded chitosan nanoparticles were evaluated by wound healing assay. As seen in Figure 6, the artificial scratch gap of control group was obviously healed, where the wound healing rates reached to 38.4% and 52.2% after incubated for 24 h and 48 h, respectively. The migration of HepG2 cells could be inhibited by free DOX, with the wound healing rates respectively reduced to 27.6% and 36.6%. Nevertheless, DOX-loaded Gly-CS-VE, Gal-Gly-CS-VE, Gly-CS-DCA, and Gal-Gly-CS-DCA nanoparticles all exhibited significantly stronger migration inhibition capabilities than free DOX (*p* < 0.05), with the wound healing rates less than 25%. It is worth noting that Gal-Gly-CS-DCA nanoparticles showed a stronger inhibitory effect on cell migration than Gly-CS-DCA nanoparticles during 48 h incubation, with a significant difference (*p* < 0.05). The results of cellular uptake, cytotoxicity, and wound healing all demonstrated that galactosylated Gal-Gly-CS-VE and Gal-Gly-CS-DCA nanoparticles had the strongest anticancer effect at the cellular level.

### 3.7. *In vivo* anticancer efficacy

**Figure 7.**
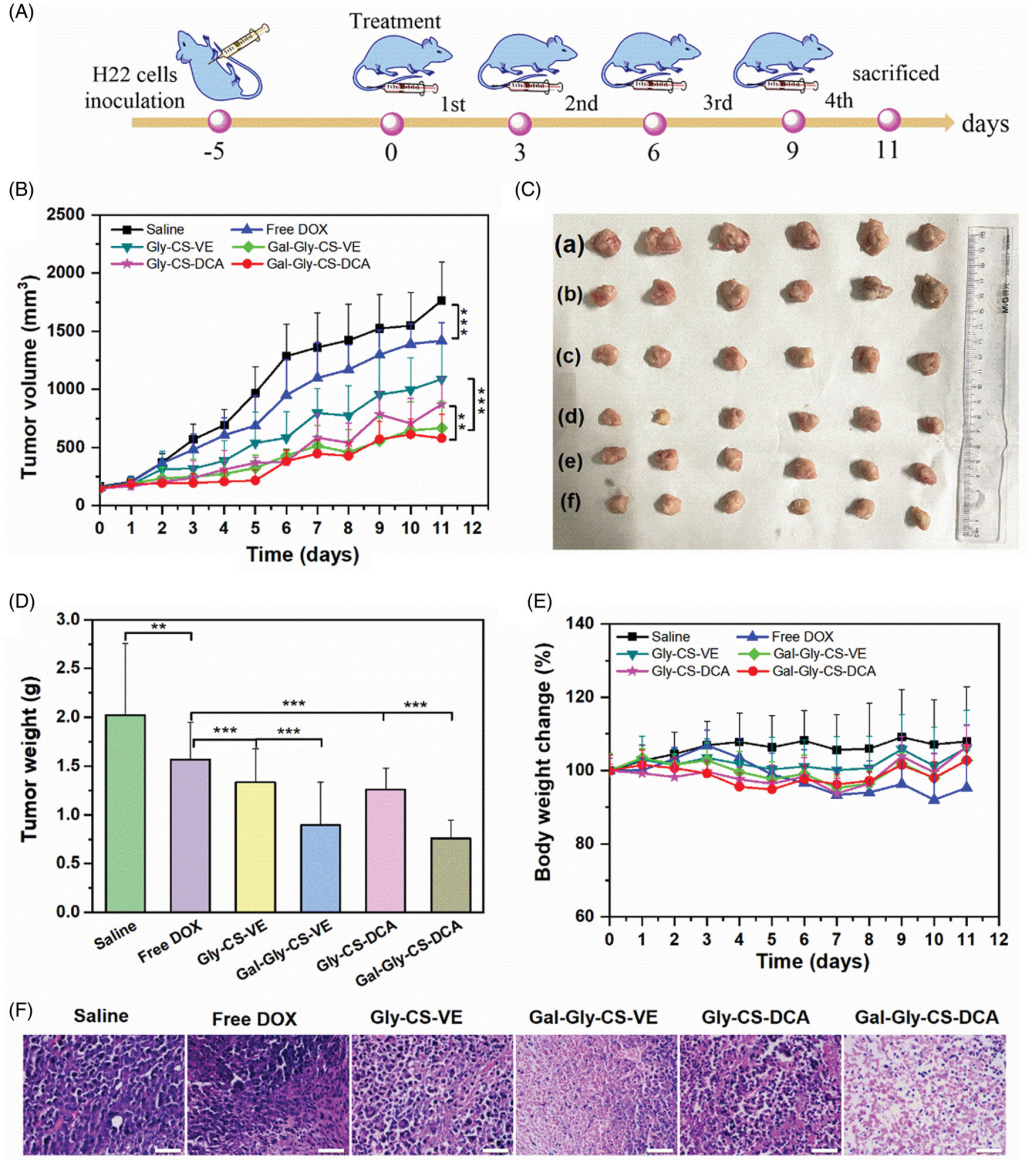
*In vivo* anticancer efficacy of DOX and DOX-loaded chitosan nanoparticles. (A) Schematic illustration of *in vivo* anticancer experimental outline. (B) Tumor volume growth curve of each group, **p < 0.01, ***p < 0.001. (C) Photographs of tumors excised from each group at Day 11: (a) Saline, (b) Free DOX, (c) Gly-CS-VE, (d) Gal-Gly-CS-VE, (e) Gly-CS-DCA, (f) Gal-Gly-CS-DCA. (D) Tumor weight of tumors excised from each group at Day 11, **p < 0.01, ***p < 0.001. (E) Body weight change curve of each group. (F) H&E staining of tumors excised from each group at Day 11, scale bar = 50 μm.

Encouraged by the apparent *in vitro* cytotoxicity of DOX-loaded chitosan nanoparticles, the *in vivo* anticancer efficacy was further evaluated by the H22 tumor xenograft model. As shown in Figure 7(A), BALB/c mice were injected with H22 cells in the right axilla, which took 5 days to form tumors with the volume of 100–150 mm^3^. Then the mice were divided into six groups, and separately treated with saline, free DOX, Gly-CS-VE, Gal-Gly-CS-VE, Gly-CS-DCA, and Gal-Gly-CS-DCA nanoparticles with the DOX dosage of 5 mg/kg. The tumor volume curves were shown in Figure 7(B), and we found the tumors of all the six groups were gradually increased over time. The saline group exhibited the most rapid increase of tumor volumes, which reached to approximately 1750 mm^3^ on Day 11. By contrast, groups containing DOX all exhibited tumor inhibition effect to some extent. Despite free DOX could significantly suppress the growth of tumors, the tumor volumes of free DOX group still reached to 1418 mm^3^ on Day 11, indicating the anticancer effect was unsatisfactory. The DOX-loaded chitosan nanoparticles groups all exhibited better anticancer capabilities than free DOX group, with the tumor volumes less than 1100 mm^3^ on Day 11. It is worth noting that the tumor volumes of Gal-Gly-CS-VE group and Gal-Gly-CS-DCA group were significantly smaller than that of Gly-CS-VE group (667 mm^3^ vs 1089 mm^3^) and Gly-CS-DCA group (580 mm^3^ vs 872 mm^3^), meaning the galactosylated nanoparticles had stronger *in vivo* anticancer potency. The appearance and weights of tumors excised from each group on Day 11 were shown in Figure 7(C,D). The results also demonstrated that the tumors treated by DOX loaded chitosan nanoparticles were significantly smaller and lighter than those treated by saline and free DOX. Among these treatment groups, the galactosylated Gal-Gly-CS-VE group and Gal-Gly-CS-DCA group exhibited the best anticancer efficacies, with the tumor weights reduced by approximately 50% in comparison with the saline group. This could be attributed to the targeting effect of galactose group to asialoglycoprotein receptor overexpressed on HCC cells. Furthermore, the excised tumors were sliced up and stained with hematoxylin-eosin (seen in Figure 7(F)). We found the saline group showed normal histological structure with large and densely arranged nuclei. For the groups of free DOX, Gly-CS-VE, Gal-Gly-CS-VE, Gly-CS-DCA, and Gal-Gly-CS-DCA, different levels of apoptosis and necrosis could be observed where the nuclei were stained pink and light. It was apparent that the areas of cell apoptosis in the tumors treated by Gal-Gly-CS-VE and Gal-Gly-CS-DCA nanoparticles were significantly larger than that treated by Gly-CS-VE and Gly-CS-DCA nanoparticles. Overall, these results fully demonstrated that DOX-loaded chitosan nanoparticles could enhance the *in vivo* anticancer efficacy of DOX. Benefiting from the ASGPR targeting effect, the anticancer capabilities of galactosylated nanoparticles (Gal-Gly-CS-VE and Gal-Gly-CS-DCA) were significantly stronger than nanoparticles without galactose residue (Gly-CS-VE and Gly-CS-DCA).

### 3.8. *In vivo* toxicity

**Figure 8.**
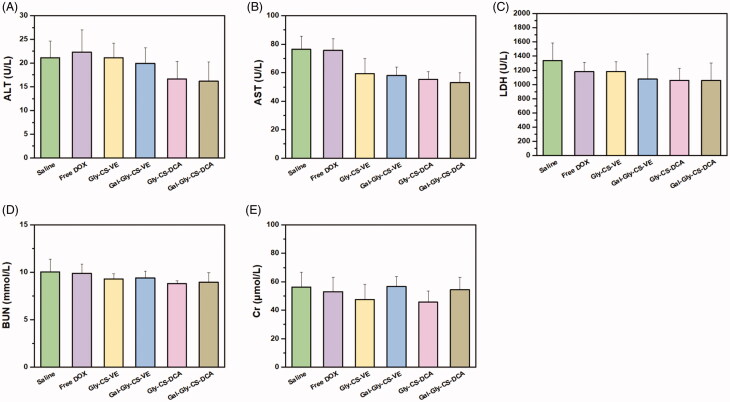
Plasma chemistry parameters of mice treated by DOX and DOX loaded chitosan nanoparticles for 11 days. (A) Alanine aminotransferase (ALT), (B) aspartate aminotransferase (AST), (C) lactate dehydrogenase (LDH), (D) blood urea nitrogen (BUN) and (E) creatine kinase (CK).

**Figure 9.**
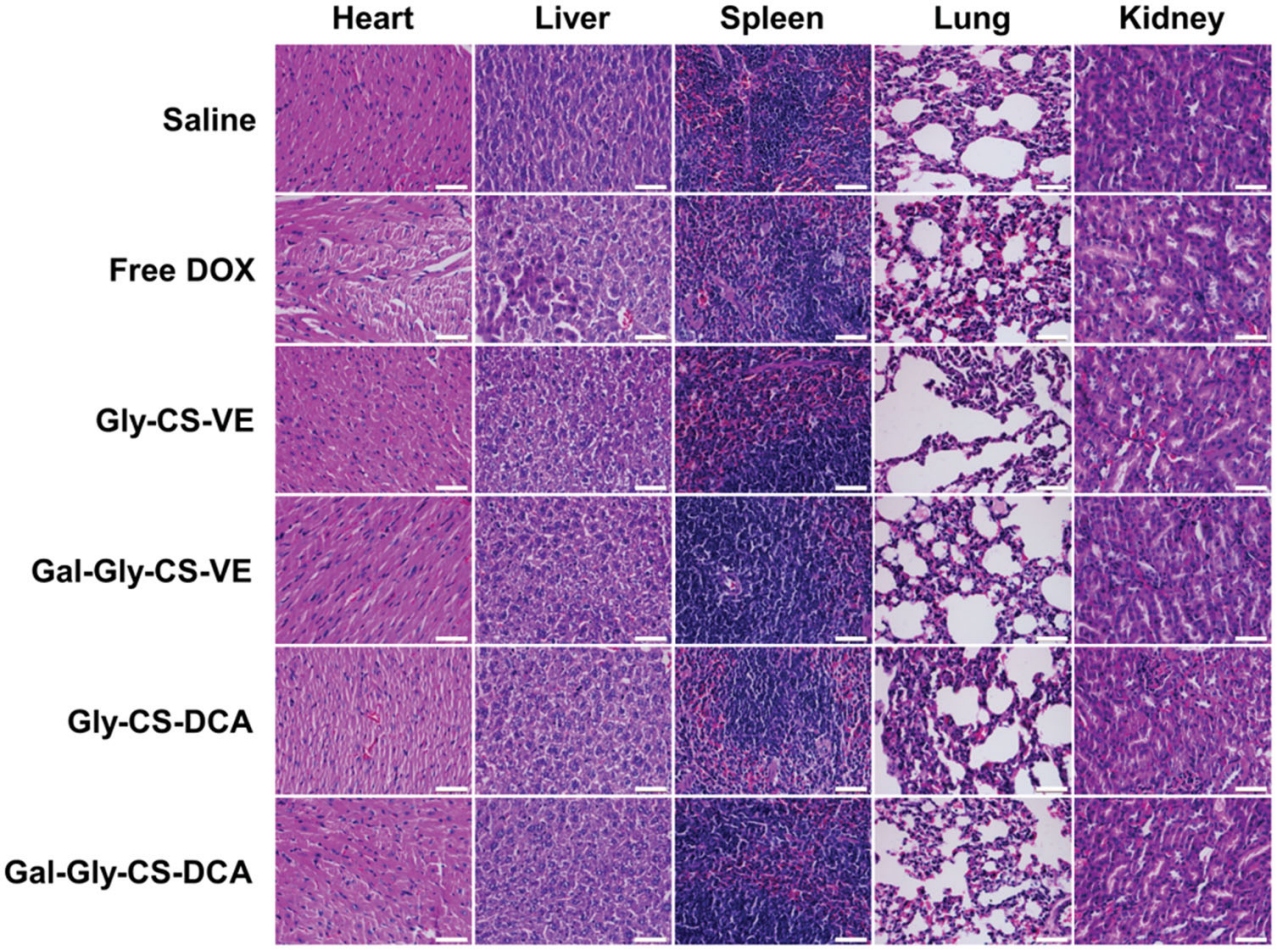
H&E staining of major organs of mice treated by DOX and DOX-loaded chitosan nanoparticles for 11 days, scale bar = 50 μm.

Besides the therapeutic effects, the biosafety is also a key consideration of drug formulation for clinical application. In this work, the *in vivo* toxicity of DOX and DOX-loaded chitosan nanoparticles were evaluated by the change of body weight, plasma chemistry parameters and major organ histopathology. As seen in Figure 7(E), no remarkable change of body weight was recorded, with the percentage of change less than 10%. There was no significant difference between the treatment groups and the saline group, implying the DOX-loaded chitosan nanoparticles had no overall side effects on mice. Furthermore, their toxicity on organs was tested by some plasma chemistry parameters including alanine aminotransferase (ALT), AST, LDH, BUN, and CK. It is well known that ALT and AST are indicators of liver damage, BUN and CK are indicators of kidney damage, while LDH could reflect the cardiotoxicity. As depicted in Figure 8, the levels of ALT, AST, LDH, BUN, and CK were similar among the six groups, with no significant difference. This indicated that DOX-loaded chitosan nanoparticles did not cause serious damage to the liver, kidney, and heart during treatment. Finally, the major organs (heart, liver, spleen, lung, and kidney) with different treatments were sliced and stained with H&E to compare the histological changes. As shown in Figure 9, no obvious pathological variations were observed in the treatment groups in comparison with the saline group, revealing the good biocompatibility of DOX-loaded chitosan nanoparticles. Taken together, these results fully confirmed that the *in vivo* bio-safety of DOX-loaded chitosan nanoparticles were excellent, which was beneficial for clinical application.

## 4. Conclusion

In summary, four types of self-assembled chitosan nanoparticles Gly-CS-VE, Gal-Gly-CS-VE, Gly-CS-DCA, and Gal-Gly-CS-DCA were successfully fabricated to load DOX for HCC therapy, where the galactosylated Gal-Gly-CS-VE and Gal-Gly-CS-DCA nanoparticles could selectively target to liver via recognizing ASGPR. The chitosan nanoparticles were spherical in shape, with the average particle sizes in the range of 150–200 nm. Remarkably, the release of DOX from four chitosan nanoparticles all performed pH-dependent manners, where DOX was released out faster in acidic condition (pH 5) than in physiological condition (pH 7). Benefiting from the ASGPR targeting effect of galactose residue, the galactosylated Gal-Gly-CS-VE and Gal-Gly-CS-DCA nanoparticles showed enhanced *in vitro* cell internalization, cytotoxicity, and anti-migration capabilities than Gly-CS-VE and Gly-CS-DCA nanoparticles. Furthermore, the Gal-Gly-CS-VE and Gal-Gly-CS-DCA nanoparticles also exhibited enhanced *in vivo* anticancer efficacies than the Gly-CS-VE and Gly-CS-DCA nanoparticles. Finally, the four chitosan nanoparticles all exhibited good biocompatibility without causing any obvious histological damage to the major organs. Overall, the galactosylated chitosan nanoparticles were proven to be promising pharmaceutical formulations for selectively overcoming HCC, with great potential for clinical applications.

## Supplementary Material

Supplemental Material
