# Supplementary material for: In vitro and in vivo evaluation of self-assembled chitosan nanoparticles selectively overcoming hepatocellular carcinoma via asialoglycoprotein receptor
Source: Drug Deliv. 2021 Oct 1;28(1):2071–84. doi: 10.1080/10717544.2021.1983077 (PMC8491732; doi:10.1080/10717544.2021.1983077)
Supplement: Supplemental Material [file IDRD_A_1983077_SM6232.docx]

***In vitro* and *in vivo* evaluation of** **self-assembled chitosan nanoparticles selectively overcoming hepatocellular carcinoma via asialoglycoprotein receptor**

Rensong Sun ^a,1^, Linlin Fang ^a,1^, Xia Lv ^b^, Jiani Fang ^a^, Yuting Wang ^a^, Dapeng Chen ^c^, Liang Wang ^c^, Jun Chen ^c^, Yan Qi ^a^, Zeyao Tang ^a^, Jianbin Zhang ^a,*^, Yan Tian ^a,*^

^a^ Collage of Pharmacy, Dalian Medical University, Dalian 116044, China.

^b^ Collage of Integrative Medicine, Dalian Medical University, Dalian 116044, China

^c^ Laboratory Animal Center, Dalian Medical University, Dalian 116044, China

^1^ Theses authors contributed equally to this work.

**^*^ Corresponding authors:**

**Jianbin Zhang,**

College of Pharmacy, Dalian Medical University, 9 West Sect Lvshun South Rd, Dalian 116044, China. E-mail: [zhangjb@dmu.edu.cn](mailto:zhangjb@dmu.edu.cn)

**Yan Tian,**

College of Pharmacy, Dalian Medical University, 9 West Sect Lvshun South Rd, Dalian 116044, China. E-mail: [tiany2004@126.com](mailto:tiany2004@126.com)

**Supplemental material**

1. **Optimization of Gly-CS-VE and Gly-CS-DCA**

The appearance and the characterizations of Gly-CS-VE obtained from different chitosan glucosamine residue/VE mole ratios were shown in **Figure S1** and **Table S1**. At the concentration of 2.5 mg/mL, we found the four kinds of nanoparticles were light blue opalescent with obvious Tyndall effect, and their particle sizes were between 150-180 nm. This indicated they were nano-sized self-assembled particles in water. As the hydrophobic core was the main position of solubilizing poorly water-soluble drugs, the higher VE proportion should be more beneficial for loading drugs. However, the 100:10 group nanodispersion was more opaque than other groups, which indicated the present of large particles. Hence, we selected the Gly-CS-VE nanoparticles from the chitosan glucosamine residue/VE mole ratio of 100:5 as the model for further study. Because of the same reason, we selected the Gly-CS-DCA nanoparticles from the chitosan glucosamine residue/DCA mole ratio of 100:30 as the model for further study (see **Figure S2** and **Table S2**).

**
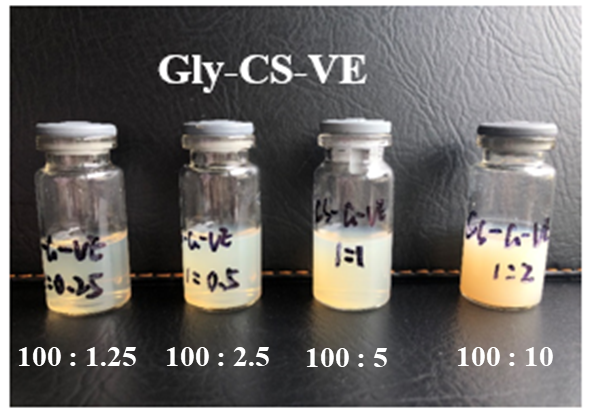
**

**Figure S1** Appearance of Gly-CS-VE nanoparticles in aqueous solution obtained from different chitosan glucosamine residue/VE mole ratios: 100:1.25, 100:2.5, 100:5 and 100:10.

**Table S1** Characterizations of Gly-CS-VE nanoparticles obtained from different chitosan glucosamine residue/VE ratios: 100:1.25, 100:2.5, 100:5 and 100:10.

| Chitosan glucosamine residue/VE mole ratio | **Size (nm)** | **PDI** | **Zeta (mV)** |
| --- | --- | --- | --- |
| 100:1.25 | 158.6±1.3 | 0.126±0.034 | 6.71±0.35 |
| 100:2.5 | 161.8±1.2 | 0.128±0.026 | 13.97±0.21 |
| 100:5 | 178.9±2.3 | 0.147±0.003 | 19.8±0.44 |
| 100:10 | 175.8±0.7 | 0.188±0.015 | 14.8±0.53 |


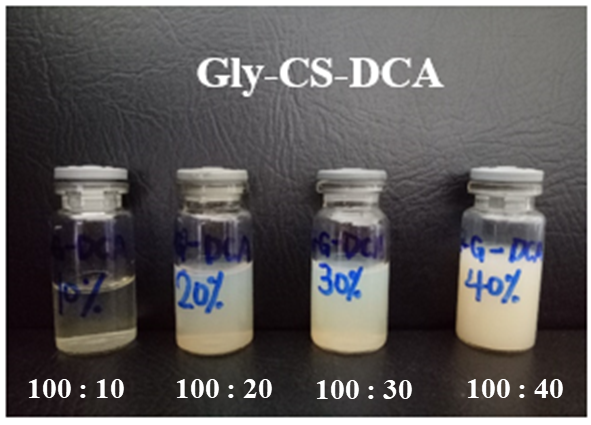


**Figure S2** Appearance of Gly-CS-DCA nanoparticles in aqueous solution obtained from different chitosan glucosamine residue/DCA mole ratios: 100:10, 100:20, 100:30 and 100:40.

**Table S2** Characterizations of Gly-CS-DCA nanoparticles obtained from different chitosan glucosamine residue/VE ratios: 100:10, 100:20, 100:30 and 100:40.

| Chitosan glucosamine residue/DCA mole ratio | **Size (nm)** | **PDI** | **Zeta (mV)** |
| --- | --- | --- | --- |
| 100:10 | 158.47±2.58 | 0.194±0.024 | 12.67±0.91 |
| 100:20 | 162.07±1.50 | 0.152±0.020 | 18.73±0.59 |
| 100:30 | 142.73±1.10 | 0.164±0.017 | 24.47±0.97 |
| 100:40 | 205.47±0.72 | 0.363±0.038 | 23.57±0.60 |

1. **Optimization of Gly-CS-VE and Gly-CS-DCA**

**Figure S3**, **Table S3** and **Table S4** showed the appearance and the characterizations of Gal-Gly-CS-VE and Gal-Gly-CS-DCA obtained from different chitosan glucosamine residue/ galactose residue mole ratios. We the graft of galactose residue on the nanoparticles did not influence their properties. As the galactose residues were the liver-targeting ligands, the higher substitution degree (DS) of galactose residues should be beneficial for the liver-targeting effect. In this work, we selected the nanoparticles from chitosan glucosamine residue/galactose residue mole ratios of 100:120 as model Gal-Gly-CS-VE or Gal-Gly-CS-VE for further study. The 100:150 groups were not selected, because the amounts of lactobionic acid of 100:120 group was high enough to react with glucosamine residue. More lactobionic acid might be wasted.

**
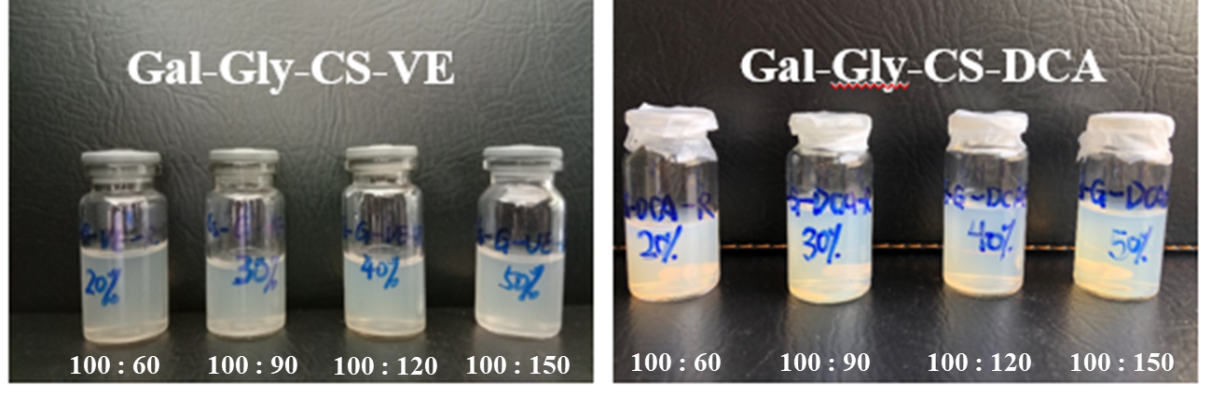
**

**Figure S3** Appearance of Gal-Gly-CS-VE and Gal-Gly-CS-DCA nanoparticles in aqueous solution obtained from different chitosan glucosamine residue/galactose residue mole ratios: 100:60, 100:90, 100:120 and 100:150.

**Table S3** Characterizations of Gal-Gly-CS-VE nanoparticles obtained from different chitosan glucosamine residue/galactose residue ratios: 100:60, 100:90, 100:120 and 100:150.

| Chitosan glucosamine residue/galactose residue ratio | **Size (nm)** | **PDI** | **Zeta (mV)** |
| --- | --- | --- | --- |
| 100:60 | 162.57±3.97 | 0.200±0.015 | 11.07±0.15 |
| 100:90 | 159.97±6.24 | 0.227±0.018 | 10.22±0.34 |
| 100:120 | 142.07±4.89 | 0.176±0.015 | 7.57±0.45 |
| 100:150 | 162.43±2.55 | 0.252±0.003 | 8.47±0.72 |

**Table S4** Characterizations of Gal-Gly-CS-DCA nanoparticles obtained from different chitosan glucosamine residue/galactose residue ratios: 100:60, 100:90, 100:120 and 100:150.

| Chitosan glucosamine residue/galactose residue ratio | **Size (nm)** | **PDI** | **Zeta (mV)** |
| --- | --- | --- | --- |
| Gal-Gly-CS-DCA 20% | 150.00±0.95 | 0.104±0.021 | 12.77±0.25 |
| Gal-Gly-CS-DCA 30% | 147.97±5.08 | 0.159±0.026 | 15.47±0.50 |
| Gal-Gly-CS-DCA 40% | 159.90±2.60 | 0.134±0.018 | 17.17±1.55 |
| Gal-Gly-CS-DCA 50% | 150.43±1.80 | 0.125±0.028 | 21.43±1.42 |
